# The mTOR signaling pathway regulates pain-related synaptic plasticity in rat entorhinal-hippocampal pathways

**DOI:** 10.1186/1744-8069-9-64

**Published:** 2013-12-09

**Authors:** Dan Lyu, Wenli Yu, Ning Tang, Ruirui Wang, Zhenyu Zhao, Fang Xie, Yongjin He, Hongyin Du, Jun Chen

**Affiliations:** 1 Department of Anesthesiology, Tianjin First Center Hospital, TianJin 300192, China; 2 Reproductive Medicine Center, General Hospital of Jinan Military Command, Jinan, Shandong 250031, China; 3 Institute for Biomedical Sciences of Pain, Capital Medical University, Beijing 100069, China; 4 Institute for Biomedical Sciences of Pain and Institute for Functional Brain Disorders, Tangdu Hospital, The Fourth Military Medical University, Xi’an 710038, China; 5 Department of Neurosurgery, General Hospital of Jinan Military Command, Jinan, Shandong 250031, China

**Keywords:** Bee venom, Hippocampal formation, Synaptic plasticity, Rapamycin, mTOR signaling pathway

## Abstract

**Background:**

Our previous work demonstrated that persistent peripheral nociception (PPN) leads to synaptic plasticity and functional changes in the rat hippocampus. The protein kinase mTOR is a critical regulator of protein synthesis-dependent synaptic plasticity in the hippocampus as well as synaptic plasticity associated with central and peripheral pain sensitization. We examined the role of mTOR signaling in pain-associated entorhinal cortex (EC) - hippocampal synaptic plasticity to reveal possible cellular mechanisms underlying the effects of chronic pain on cognition and emotion.

**Results:**

Subcutaneous injection of bee venom (BV) into one hind paw to induce PPN resulted in sustained (> 8 h) mTOR phospho-activation and enhanced phosphorylation of the mTOR target p70 S6 kinase (S6K) in the hippocampus. The magnitude and duration of long-term potentiation (LTP) in both EC - dentate gyrus (DG) and EC - CA1 synaptic pathways were elevated in BV-treated rats as measured by microelectrode array recording. Moreover, the number of potentiated synapses in the hippocampus was markedly upregulated by BV-induced PPN. Both elevated mTOR-S6K signaling and enhanced LTP induced by BV injection were reversed by systemic injection of the mTOR inhibitor rapamycin (RAPA). Rats injected with BV exhibited markedly reduced ambulation and exploratory activity in the open field (signs of depression and anxiety) compared to controls, and these effects were also reversed by RAPA.

**Conclusion:**

We suggest that PPN-induced enhancement of synaptic plasticity in EC - hippocampal pathways and the behavioral effects of PPN are dependent on mTOR-S6K signaling.

## Background

Pain is a complex sensory modality involved in sensory discrimination, affective motivation, and cognitive evaluation [1]. As a homeostatic mechanism, nociception serves to protect against injury. However, persistent nociceptive stimuli may develop into chronic pain, a neurological condition that greatly reduces quality of life. Nociceptive signals are transmitted along ascending pathways from the spinal cord to many regions of the brain involved not only in sensory perception but also in emotion and cognition. Thus, chronic pain can disrupt many higher brain functions and lead to cognitive disorders and mental illness.

Sensitization of primary sensory neurons and altered synaptic plasticity in the spinal dorsal horn contribute to chronic pain [2,3]. In contrast, the effects of chronic nociceptive input on the structure and function of higher brain structures are still largely unexplored, greatly impeding progress in clinical pain management and development of more efficacious analgesics and other pain therapies. Revealing the cellular and molecular signaling mechanisms mediating the effects of chronic pain on pain perception, emotion, and cognition will improve our understanding of chronic pain development and provide insight into therapeutic strategies for relieving the negative emotional responses to chronic pain [4].

Chronic pain alters synaptic transmission in the cerebral cortex and limbic system [5,6]. The hippocampal formation is an integral part of the Papez circuit involved in learning, memory, emotion, and motivation [7]. Functional magnetic resonance imaging, electrophysiological, and neurochemical studies have demonstrated that formalin-induced nociception can alter hippocampal neuron activity, as well as the expression and activation of proteins in these neurons [8]. Intrahippocampal injection of NMDA receptor antagonists [9] attenuates these changes, strongly implicating hippocampal processing of nociceptive information in the deleterious effects of chronic pain on cognitive, emotional, and motivational functions [8]. Nonetheless, the impacts of chronic pain on hippocampal structure and function are still rarely examined.

Target of rapamycin (TOR) was first described in TOR1 and TOR2 *Saccharomyces cerevisiae* mutants [10]. The *TOR1* and *TOR2* genes encode homologous (60%) TOR1 and TOR2 proteins of 280 kDa. The highly conserved mammalian homologue mTOR is a member of the phosphoinositide 3-kinase related kinase (PIKK) family of serine/threonine kinases [11]. Rapamycin is a lipophilic antibiotic isolated from a strain of *Streptomyces hygroscopicus* indigenous to Easter Island (Rapa Nui) that is currently used as an immunosuppressive drug [12]. The eukaryotic rapamycin receptor is the 12 kDa FK506-binding protein 12 (FKBP12). A rapamycin-FKBP12 "gain-of-function" complex interacts specifically with mTOR to potently inhibit signaling to downstream targets [13]. The mTOR signaling pathway regulates transcription, initiation of translation, ribosome biosynthesis, and apoptosis in response to multiple activating factors [14]. However, whether the mTOR signaling pathway is involved in the regulation of pain and hyperalgesia is unresolved.

Recent studies demonstrated that mTOR regulates cell growth, proliferation, and synaptic plasticity by activating protein synthesis [14]. Many downstream mTOR effector proteins are crucial for translation, including eIF4E binding protein-1 (4EBP1), ribosomal protein S6 kinase (p70S6K), and eukaryotic elongation factor 2 (eEF2) [15]. To regulate cell growth and proliferation, mTOR phosphorylates 4EBP1 and S6K to initiate translation. Phosphorylation of eEF2, a protein regulating polypeptide elongation and ribosomal translocation, is indirectly regulated by mTOR. Rapamycin blocks long-term potentiation (LTP) in the rat hippocampus induced by either tetanic stimulation or BDNF [16]. Medina et al. also demonstrated that mTOR signaling participates in hippocampus-dependent long-term memory consolidation [17]. It is established that long-lasting synaptic plasticity, neurite development, and response to injury are regulated by local protein synthesis in axons and dendrites [18], which contain all necessary components for local protein synthesis, including mRNAs and ribosomes [19]. The contribution of mTOR signaling to local protein synthesis is currently a major research focus. Jimenez-Diaz et al. reported that mTOR-dependent local mRNA translation maintained hyperalgesia mediated by A-fiber nociceptors [20]. In addition, mTOR participates in central and peripheral nociceptive sensitization [21]. Recently, Asante et al. revealed a role for mTOR in persistent pain maintenance mediated by mRNA translation and local protein synthesis following formalin-induced nociception [22].

Here, we used bee venom (BV) injection, a reliable model of persistent peripheral nociception (PPN) [23], to investigate the role of mTOR in structural and functional changes in the hippocampus induced by pain. We examined molecules activated by the mTOR signaling pathway, the effect of mTOR signaling on synaptic plasticity at DG and CA1 synapses, and the effects PPN-induced mTOR activation on behavior.

## Results

### Persistent peripheral nociceptive stimuli activated the mTOR signaling pathway in rat hippocampus

**Figure 1:**
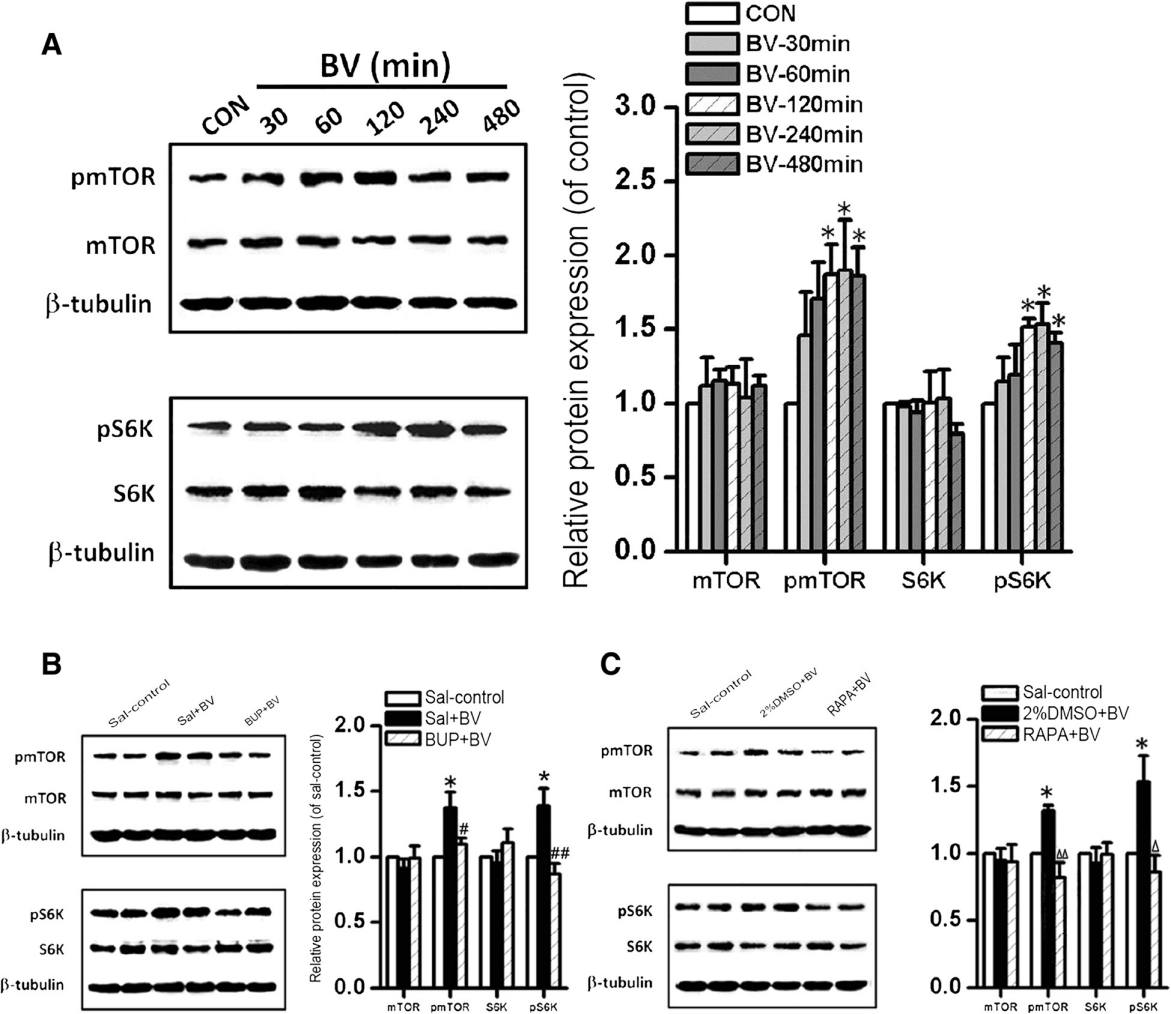
**Activation of the mTOR signaling pathway in the hippocampus by persistent peripheral nociception, but the effect was reversed by rapamycin (RAPA). (A)** Western blot analysis of p-mTOR, mTOR, p-S6K, and S6K expression in hippocampal lysates at various times after bee venom (BV) treatment. **(B)** Western analysis of p-mTOR, mTOR, p-S6K, and S6K in hippocampus tissues extracts from saline control (sal-control), Sal + BV, and bupivacaine (Bup) + BV groups. **(C)** Western blot analysis of p-mTOR, mTOR, p-S6K, and S6K in hippocampal lysates from Sal-control, 2%DMSO + BV, and RAPA + BV groups. Relative expresions of p-mTOR, mTOR, p-S6K, and S6K immunolabeling are shown relative to the control or sal-control (100%). β-tubulin was used as an internal control. The data were showed as means ± SD (n = 3). ^*^P < 0.05 *vs.* sal-control group; ^#^*P* < 0.05, ^##^*P* < 0.01, bupivacaine + BV group *vs.* Sal + BV; ^Δ^*P* < 0.05, ^ΔΔ^*P* < 0.01, RAPA + BV group *vs.* 2%DMSO + BV group.

The phosphorylation status of mTOR and its downstream signaling molecule S6K were significantly elevated in the hippocampus 2 h after BV injection, and phosphorylation was maintained for at least 8 h post-injection compared to the control group (p-mTOR-BV/p-mTOR-NS: 1.87 ± 0.21 at 2 h, 1.90 ± 0.34 at 4 h, 1.86 ± 0.20 at 8 h; all *P* < 0.05 *vs.* control group; p-S6K-BV/p-S6K-NS: 1.52 ± 0.05, 1.53 ± 0.14, and 1.40 ± 0.07; all *P* < 0.05 *vs.* control group) (Figure 1A). In contrast, relative expression levels of total mTOR and S6K were not significantly changed. These elevations in mTOR and S6K phosphorylation were reversed when BV injection was preceded by bupivacaine (Bup, Figure 1B) or rapamycin (RAPA, Figure 1C). The relative levels of p-mTOR and p-S6K were significantly reduced by bupivacaine pretreatment compared to matched controls (Sal + BV groups) (p-mTOR: 1.10 ± 0.05 *vs.* 1.38 ± 0.12, *P* < 0.05; p-S6K: 0.87 ± 0.08 *vs.* 1.39 ± 0.13, *P* < 0.01). Similarly, rapamycin pretreatment reduced relative p-mTOR and p-S6K expression levels compared to 2% DMSO + BV controls (p-mTOR: 0.82 ± 0.11 *vs.* 1.32 ± 0.04, *P* < 0.01; p-S6K: 0.86 ± 0.13 *vs.* 1.53 ± 0.19, *P* < 0.05).

### mTOR signaling mediated changes in hippocampal synaptic plasticity induced by persistent peripheral nociceptive stimuli

The basal strength of excitatory transmission and LTP induced by theta-burst stimulation (TBS) were measured using a 8 × 8 MED array in response to perforant pathway (PP) stimulation at the hippocampal sulcus. Two types of potentials were recorded, a positive inflection in the DG and a negative inflection from the CA1. Many hippocampal excitatory pathways exhibit robust and stable LTP. Three stimulus protocols are commonly used to induce LTP, high-frequency stimulation (HFS), TBS, and primed burst stimulation. We chose TBS as it partially replicates the stimulus pattern of the naturally occurring theta rhythm recorded in the hippocampus *in vivo* during learning and memory [24].

**Figure 2:**
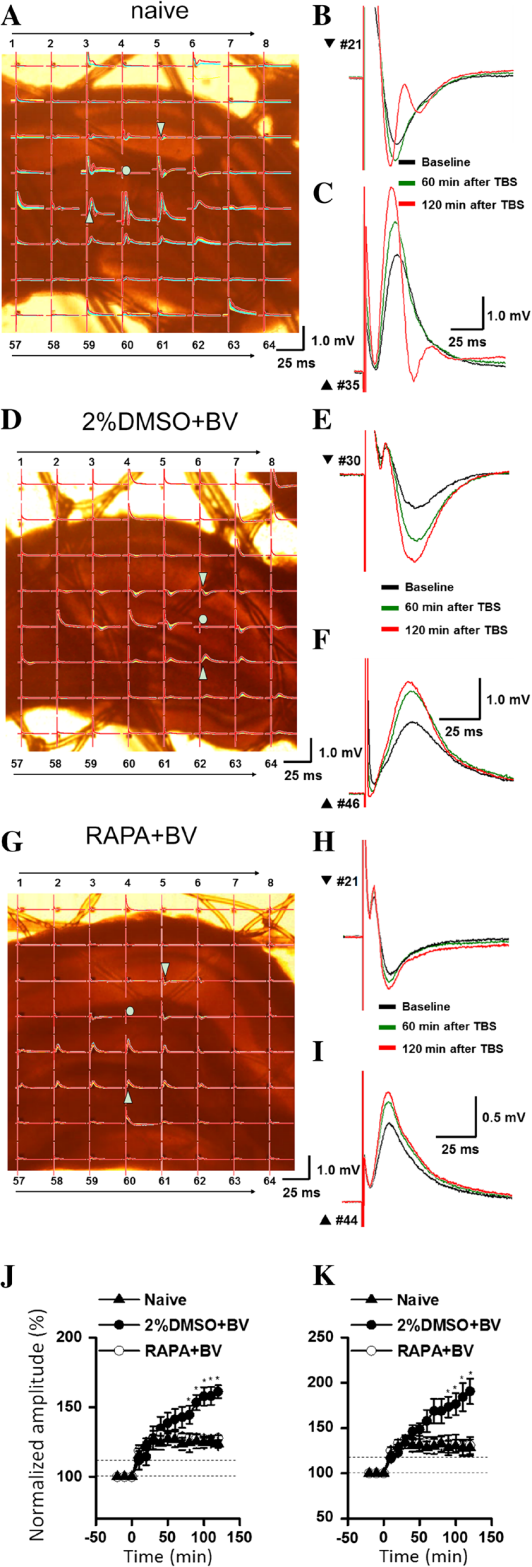
**Multichannel recording of field excitatory postsynaptic potentials (fEPSPs) in hippocampal slices from 2%DMSO + BV and RAPA + BV groups before and after theta burst stimulation. (A-C)** Responses from a naive group rat **(A)** Photograph showing the hippocampal formation from a naïve group rat and Med-64 probe with 8 × 8 array. The asterisk indicates an electrode selected for electrical stimulation of the perforant path (PP) fibers. Real traces of 63 recording electrodes across the DG and the CA1 area in response to perforant path (PP) test stimulation before (black), 60 min after (green), and 120 min (red) after TBS. The PP-evoked network FP responses were localized in both the DG and CA1. **(B)** fEPSPs are negative-going (lower) in CA1. **(C)** fEPSPs are positive-going (upper) in the DG. fEPSP amplitudes in these two areas were potentiated by TBS stimulation of PP fibers (asterisk). Scale bar applies to both sweep sets. **(D-F)** Micrograph of a hippocampal slice and evoked responses from a 2%DMSO + BV group rat. **(G-I)** Hippocampal slices and typical sample recordings from a RAPA + BV-treated rat. **(J and K)** Summary of LTP experiments in hippocampal slices from naive, 2%DMSO + BV and RAPA + BV groups. The amplitude of both the DG **(J)** and CA1 **(K)** fEPSPs was normalized to pre-TBS baseline. The data were showed as means ± SD (n = 7). ^*^P < 0.05, RAPA + BV group *vs.* 2%DMSO + BV group.

The sites of PP stimulation and recordings by the MED-64 array are shown for a naïve group rat slice (Figure 2A), 2%DMSO + BV group rat slice (Figure 2D), and RAPA + BV group rat slice (Figure 2G). Typical sample fEPSPs evoked at 50% of maximum amplitude from the DG (negative inflections) before TBS, 60 min post-TBS, and 120 min post-TBS are superimposed (Figure 2B, naïve; Figure 2E, 2%DMSO + BV; Figure 2H, RAPA + BV). Typical sample fEPSPs from the CA1 (positive inflections) evoked at 50% of maximum amplitude are also shown (Figure 2C, F, and I, respectively), The post-TBS fEPSPs were significantly larger in the 2%DMSO + BV group compared to the naïve group and the RAPA + BV group in both DG and CA1, and LTP in both pathways lasted for more than 2 hours in the 2%DMSO + BV group. The time course of LTP for the different treatment groups is summarized in Figure 2J (DG) and Figure 2K (CA1). At 2 h post-TBS, the normalized mean fEPSP amplitude in the DG was significantly greater in the 2%DMSO + BV group than in the RAPA + BV group (161.09 ± 4.60% *vs.* 127.87 ± 2.14%, n = 6 for both groups, Figure 2J). Similarly, mean LTP amplitude in the CA1 at 2 h post-TBS was significantly larger in the 2%DMSO + BV group than in the RAPA + BV group (190.56 ± 14.00% *vs.* 130.77 ± 9.32%, n = 6 for the 2%DMSO + BV group and n = 7 for the RAPA + BV group, Figure 2K). Moreover, mean DG and CA1 LTP amplaitudes in RAPA + BV group slices were not significantly different from naïve rat slices, indicating complete reversal of the pain-mediated LTP enhancement. The amplitude of LTP started to decrease 80 min (DG) and 90 min (CA1) after TBS in the RAPA + BV group, while the magnitude continued to increase for the entire 2 h recording period in the vehicle-treated group.

### mTOR signaling regulated the spatial extent of synaptic plasticity induced by persistent peripheral nociceptive stimuli

**Figure 3:**
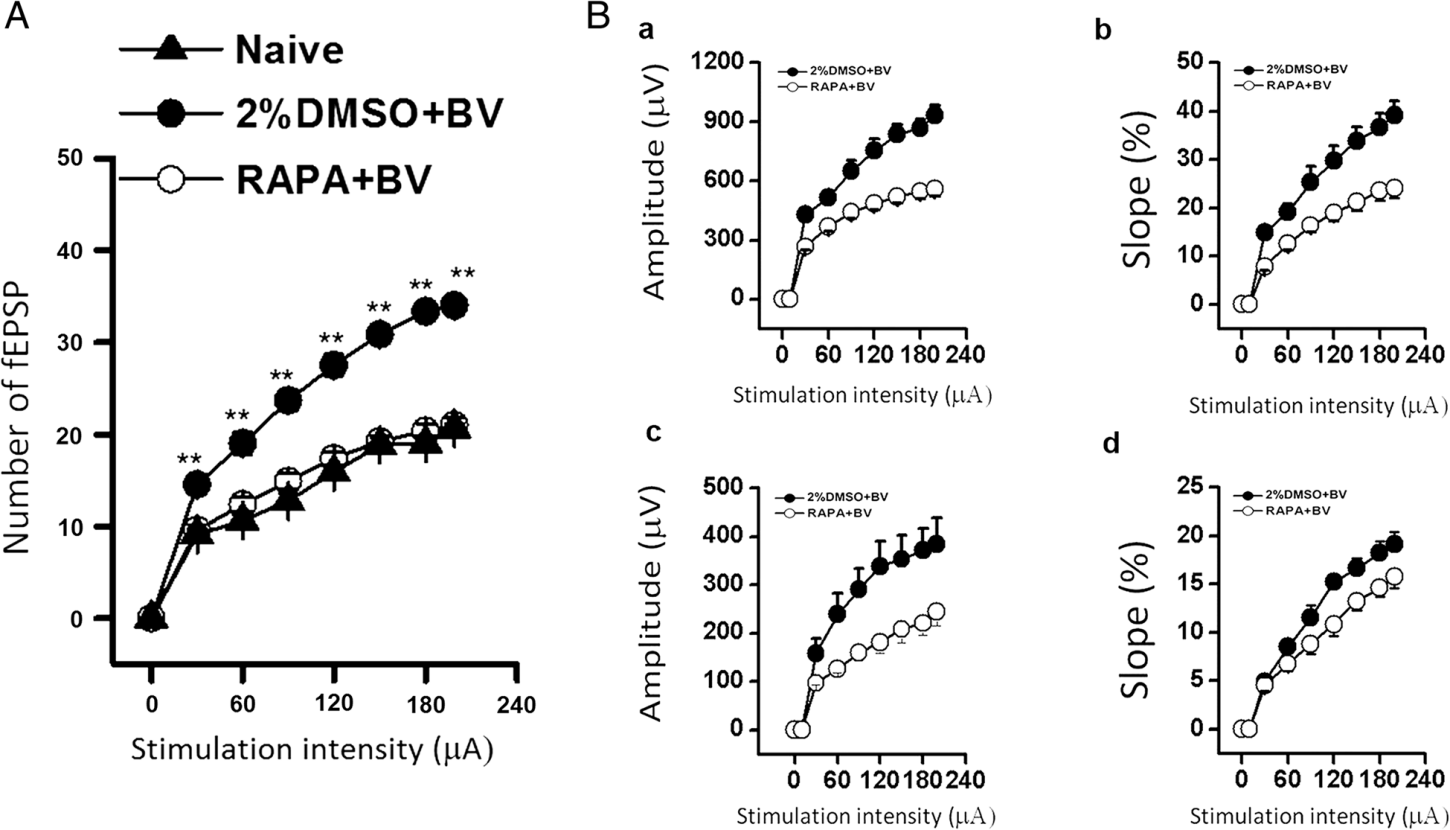
**Spatial distribution of pain-potentiated synapses in hippocampal slices from naive, 2%DMSO + BV and RAPA + BV groups. (A)** Averaged number of fEPSPs reliably evoked at >20% baseline ("large" fEPSPs) across the 8 × 8 array with increasing stimulus intensity. Compared to the 2%DMSO + BV group, the number of large fEPSPs in the RAPA + BV treated group was significant lower at each suprathreshold stimulus applied (30 - 199 μA). **(B)** Stimulus intensity-network response function curves in slices from 2%DMSO + BV and RAPA + BV groups. Individual fEPSP in DG (upper) and CA1 (lower) area increased in amplitude (a, b) or slope (c, d) at greater stimulus intensity. The input-output (I-O) function curves of the hippocampal network response were right-shifted in the RAPA + BV treatment group compared to the 2%DMSO + BV treatment group. The data were showed as means ± SD (n = 8). ^*^P < 0.05, ^**^P < 0.01 RAPA + BV group *vs.* 2%DMSO + BV group.

The number of channels in which the mean fEPSP induced by stimulus pulses from 30 to 199 μA was > 20% of the baseline fEPSP amplitude at the channel chosen to record LTP (Figure 3A) was used as an index of the spatial distribution of pain-induced synaptic potentiation. Stimulus intensity-network response function curves for both DG and CA1 are presented in Figure 3A, and input/output curves (amplitude and slope of fEPSPs against stimulus intensity) for both DG and CA1 recording sites are shown in Figure 3B. The amplitudes of the DG and CA1 potentials increased as stimulus intensity increased, and the fEPSP slope in DG and CA1 displayed a linear relationship with increasing stimulus intensity. Compared to the 2%DMSO + BV group, the stimulus intensity-network response function curve of the naïve and RAPA + BV groups exhibited a shallower rise (Figure 3A, B). In other words, the number of channels in which fEPSP amplitude was > 20% of the principal response channel baseline was lower in naïve and RAPA + BV group rats than 2%DMSO + BV group rats at the same stimulus intensity, suggesting a more restricted spatial distribution of strong pain-induced synaptic potentiation (30 μA: 9.63 ± 0.56 vs. 14.50 ± 0.56, 60 μA: 12.38 ± 0.75 vs. 19.00 ± 1.21, 90 μA: 14.88 ± 0.83 vs. 23.67 ± 1.15, 120 μA: 17.38 ± 0.68 vs. 27.50 ± 1.18, 150 μA: 19.25 ± 0.59 vs. 30.83 ± 0.60, 180 μA: 20.38 ± 0.73 vs. 33.33 ± 0.67, 199 μA: 21.00 ± 0.85 vs. 34.00 ± 0.68; n = 6 for all). Our results indicate that rapamycin inhibits the tendency for chronic pain to enhance glutamatergic synaptic transmission over a broad area of the hippocampus. Conversely, unsuppressed chronic pain may increase the mean activation area of the hippocampus in response to a given EC input.

### mTOR signaling regulated changes in spontaneous locomotor activity induced by persistent peripheral nociceptive stimuli

**Figure 4:**
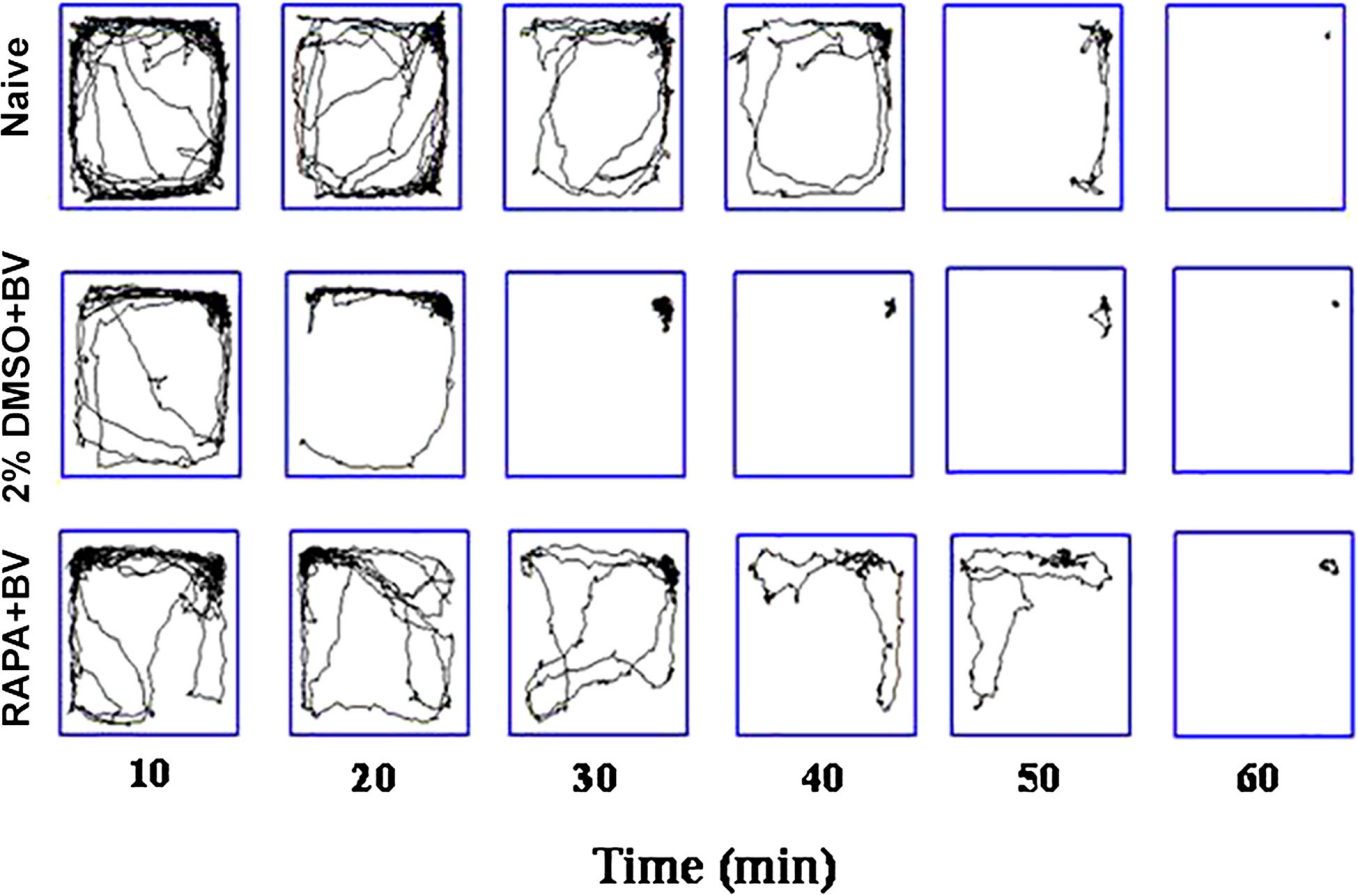
**Locomotion tracks in the open field (OF) were recorded in 10-min blocks and the preferred place data collected over 1 hour.** Locomotion decreased in all groups (naive group without any treatment; RAPA + BV group; 2%DMSO + BV group) over 1 hour, but was lowest in the 2%DMSO + BV group.

**Figure 5:**
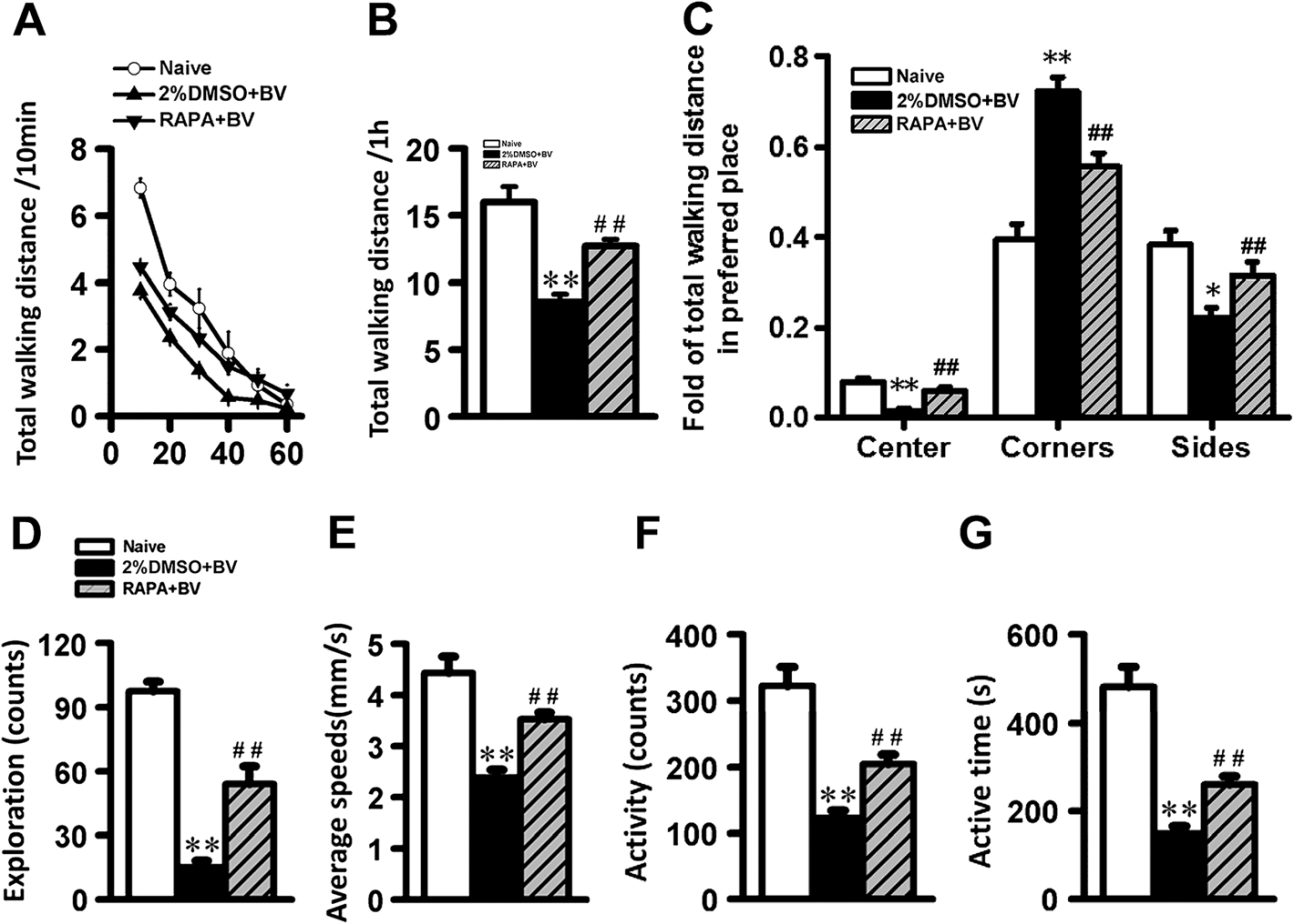
**The total distance and region-specific distances traveled in the OF by naive, 2%DMSO + BV, and RAPA + BV groups. (A)** The total distance travelled in the OF after 10, 20, 30, 40, 50 and 60 min. **(B)** The total distance over an entire hour. **(C)** The fold of distance traveled in the center, corner, or side regions of the OF to the total distance traveled (an index of place preference) for naive, 2%DMSO + BV, and RAPA + BV groups. **(D - G)** Differences in **(D)** number of hole explorations, **(E)** average speed, **(F)** number of locomotor events, and **(G)** time spent in locomotion for naive, 2%DMSO + BV, and RAPA + BV groups. The data were showed as means ± SD (n = 10). ^*^P < 0.05, ^**^P < 0.01 *vs.* naïve group; ^#^*P* < 0.05, ^##^*P* < 0.01 RAPA + BV group *vs.* 2%DMSO + BV group.

Changes in spontaneous locomotor activity were assessed in the open field (OF). The hippocampal formation is an integral part of the Papez circuit that regulates emotion and motivation, so changes in synaptic strength and plasticity may alter spontaneous activity. Parameters recorded in the OF for the naïve group, 2%DMSO + BV group, and RAPA + BV group were trajectory (Figure 4), total distance travelled, distance travelled in the central, peripheral, and corner zones, number of hole explorations, average speed, activity counts, and duration of activity (all in Figure 5). Movement diminished with time for all three groups, but 2%DMSO + BV group rats exhibited the shortest total distance travelled during 1 h in the OF. These rats often remained at a fixed point in one of the OF corners for 30 min or longer. The ratios of total distance in the central and peripheral regions to total distance travelled were significantly lower in the 2%DMSO + BV group, while the ratio of total distance in the four corner regions to total distance travelled was higher in the 2%DMSO + BV group (Figure 4). Thus, 2%DMSO + BV-treated rats exhibited reduced ambulation, a behavioral endophenotype of depression. Moreover, increased time spent in the corners is indicative of greater anxiety in 2%DMSO + BV rats. These signs of depression and anxiety were reversed by RAPA pretreatment. Finally, the number of hole explorations, average speed, number of mobility events, and the duration of these events were all significantly lower in 2%DMSO + BV group rats compared to naïve and RAPA + BV group rats (Figure 5). Based on the reversal of these effects by RAPA, we suggest that mTOR signaling is involved in the emotional response of rats to PPN.

## Discussion

In 1937, Papez proposed that the limbic system was the anatomic region underlying emotion (and so is often called the Papez circuit). The hippocampus and associated structures of the medial temporal lobe are integral parts of the Papez circuit and critical structures for learning and memory [25,26]. Recent evidence also supports the involvement of the hippocampus and EC in pain processing [27]. Melzack and Casey suggested in 1968 that the limbic system, including the hippocampus, plays a vital role in the formation of motivational drive and negative emotional responses to pain [15], but the precise cellular and molecular mechanisms have remained elusive. Previous work from our laboratory demonstrated altered temporal synaptic plasticity and transmission in EC - hippocampal pathways induced by persistent peripheral nociceptive stimuli [28], and the current study implicates hippocampal mTOR signaling in both altered hippocampal plasticity and the behavioral responses to persistent pain.

Dendritic protein synthesis is critical for long-lasting synaptic modifications [29], and mTOR is thought to modulate the local translation of mRNAs via the phosphorylation of several downstream translation effector proteins, including S6K1 and 4EBP1 [30]. Phosphorylation of S6K1 mediates translation initiation and polypeptide elongation, while 4EBP1 is a repressor of eukaryotic initiation factor 4E (eIF4E). Interaction of 4EBP1 with eIF4E results in specific inhibition of cap-dependent translation [30]. Activation of mTOR signaling by postsynaptic receptors and ion channels [31,32] induces the dendritic protein synthesis required to maintain long-lasting forms of synaptic plasticity, particularly late-LTP (L-LTP). This study suggests that persistent peripheral pain also activates the mTOR pathway in hippocampal dendrites, thereby lowering the threshold or increasing the strength of TBS-induced L-LTP.

There are two behaviorally relevant aspects of synaptic plasticity, changes in strength over time and the spatial distribution of these changes. Hippocampal LTP is a form of activity-dependent synaptic plasticity widely studied as a cellular model of associative learning, most notably at EC - DG granule cell synapses and EC - CA1 pyramidal cell synapses [33]. Theoretically, enhanced synaptic strength could result from greater presynaptic glutamate release (through release of greater numbers of quanta per bouton or the recruitment of greater numbers of presynaptic axons) or an enhanced postsynaptic response to the same level of glutamate release. These opposing mechanisms have been debated for several decades [33].

In contrast, the spatial distribution of these changes in synaptic strength has been studied less extensively. Here, we explored these changes during persistent peripheral nociceptive stimuli using a planar microelectrode array. Previous work has successfully applied the MED64 probe (Panasonic, Japan) to multiple brain regions responsible for nociception [28] and confirmed its stability and reliability [34]. Persistent peripheral nociceptive stimuli can alter both spatial and temporal plasticity in the EC - hippocampal pathway [28]. Given that the mTOR signaling pathway is activated by PPN and is a known regulator of LTP, we examined the effects of mTOR inhibition on both temporal and spatial changes in synaptic strength at EC - DG and EC - CA1 synapses simultaneously using the MED 8 × 8 grid. The normalized fEPSP amplitude and LTP amplitude 2 h post-TBS (L-LTP) were significantly lower in RAPA-pretreated, BV-injected rats compared to BV-injected rats pretreated with the vehicle DMSO (2%DMSO + BV group). The maintenance of LTP is dependent on protein synthesis [35,36], and mTOR signaling is involved in local protein synthesis required for the maintenance of LTP at both EC - DG and EC - CA1 synapses. Here we show that pain-dependent mTOR activation in the hippocampus induces widespread changes in synaptic strength. In contrast to the localized changes in synaptic strength responsible for associative learning, this broadly distributed LTP may disrupt learning and other hippocampal functions.

Our previous work demonstrated that BV induced two forms of spatial synaptic plasticity, enhanced presynaptic release, probably by recruiting additional presynaptic fibers, and enlargement of the responsive hippocampal network, possibly due to formation of new synapses [28]. Input/output curves and stimulus intensity-network response functional curves were right-shifted in RAPA-treated, BV-injected rats compared to the 2%DMSO + BV group (Figure 3), indicating fewer potentiated synapses within the recording array. Synapse formation and structural alterations in dendritic spines associated with LTP are dependent on local protein synthesis [29,35,36]. In addition, systemic inhibition of mTOR blocks hyperalgesia in animal models of neuropathic pain [37-39], We suggest that mTOR-mediated local protein synthesis participates in enhancing synaptic strength and synaptogenesis to enlarge the hippocampal neural network responsive to nociceptive inputs. Taken together, mTOR signaling likely plays an important role in spatiotemporal plasticity in EC - hippocampal pathways during persistent peripheral nociceptive stimuli.

Spontaneous activity in the open field test is used to evaluate the characteristics of affective disorders induced by chronic pain, such as anxiety and depression [40,41]. Spontaneous activity, including both locomotion and exploratory behavior, was abated after injecting BV into the plantar surface of left hind paw, and these behavioral signs of anxiety and depression were reversed by rapamycin pretreatment. Thus, hippocampal mTOR signaling is likely involved in both the synaptoplastic and behavioral responses to PPN, although more specific tests of the behavioral endophenotypes of depression and anxiety are needed to strengthen this association.

Given that rapamycin was administered systemically, we cannot exclude the contribution of PPN-induced and mTOR-dependent synaptic plasticity in other brain regions to these behavioral changes. Indeed, synaptoplastic changes or neural disinhibition in the thalamus, somatosensory cortex, amygdala, insula, and (or) cingulate cortex may also contribute [42,43]. However, it should be noted that intracerebral or intrahippocampal injection can damage local circuits and will result in a sizeable drug concentration gradient, with possible non-specific effects due to excess rapamycin near the electrode tip and insufficient LTP blockade at distant synapses. Furthermore, our in vitro studies indicate that mTOR inhibition markedly reduces L-LTP in the hippocampus, and many studies implicate the hippocampus in pain processing [5,6,16,28].

## Conclusions

Persistent peripheral pain triggered prolonged activation of the mTOR signaling pathway in the hippocampus as evidenced by mTOR and S6K phosphorylation, and this response was ameliorated by rapamycin pretreatment. Rats subjected to PPN demonstrated enhanced and more widespread LTP in EC - DG and EC - CA1 pathways, and these effects were also reversed by rapamycin. Finally, rats subjected to PPN showed behavioral signs of depression and anxiety, responses which again were reversed by rapamycin. Collectively, these results strongly suggest that activation of mTOR by PPN induces dendritic protein synthesis, which in turn enhances long-lasting LTP nonspecifically and broadly throughout EC - hippocampal networks. These synaptoplastic changes alter rat behavior, although the scope of these behavioral changes and the underlying mechanisms remain to be determined.

## Methods

### Animals

Adult male Sprague-Dawley rats (body weight 180 - 240 g, age 6 - 8 weeks) were obtained from the Experimental Animal Center of the Fourth Military Medical University (Xi’an, China) and housed with ad libitum access to food and water under a 12:12 h light/dark cycle at 22 - 26°C and 50 ± 5% humidity. All procedures and animal experiments were approved by the animal Ethical Committee of the Fourth Military Medical University and all procedures were in accordance with the International Association for the Study of Pain (IASP) guidelines [44].

### Study design

A control group of rats received no treatment and are referred to as the naive group. In experimental rats, the mid-plantar surface of the left hind paw was subcutaneously injected with normal saline (NS, CON) or bee venom (BV) (0.2 mg in 50 μl NS, Sigma, MO, USA) [45]. To evaluate the analgesic effects of bupivacaine and rapamycin against BV injection, four other groups of rats were treated as described below. Bupivacaine (0.25%, 600 μl; Shanghai Harvest Pharmaceutical Co., Ltd., China) or an equal volume of NS was injected into the ankle of the left hind paw 10 min before BV administration, and BV was allowed to activate nociceptive afferents for 2 h (Bup + BV group and Sal + BV group). For rapamycin (RAPA) (Chengdu YATU biotechnology CO., LTD., China) treatment and matched RAPA control groups, rats received either 2%DMSO (Sigma, MO, USA) or RAPA (10 mg/kg RAPA in 2%DMSO) by gavage for 3 days (1 ml/d), followed 1 h after the last gavage administration by BV injection to the plantar surface of the left hind paw (2%DMSO + BV and RAPA + BV groups). In a subgroup of these rats, brains were collected 0.5, 1, 2, 4, and 8 h after injection for LTP experiments and Western blotting as described below.

### Western blotting

Rats were sacrificed by decapitation at various times after drug treatment. The hippocampus was quickly dissected from the brain, immersed in liquid nitrogen for frozen grinding, and lysed in ice-cold RIPA lysis buffer (Beyotime, Jiangsu, China). Homogenates were then centrifuged at 12000 × *g* for 10 min at 4°C and a supernatant sample retained to measure total sample protein concentration using a BCA protein assay kit (Thermo, IL, USA). The remaining supernatant samples were stored at -70°C until use.

Equal amounts of protein (30 μg) were applied to each gel lane, separated by 8% SDS-PAGE, and electrotransferred to nitrocellulose membranes (Millipore, MA, USA). Membranes were blocked with 5% milk powder (Bio-rad, CA, USA) in PBS with 0.05% Tween 20 (PBST) for 1 h at room temperature (RT), then incubated overnight at 4°C in PBST containing rabbit anti-mTOR (1:1000, Cell Signaling Technology, MA, USA), rabbit anti-p-mTOR (1:500, Cell Signaling Technology), rabbit anti-S6K (1:500, Cell Signaling Technology), rabbit anti-p-S6K (1:500, Cell Signaling Technology), and mouse anti-β-tubulin (1:10000, Sigma, MO, USA). Membranes were then washed three times (10 min/wash) with PBST and incubated for 1 h at RT with a horseradish peroxidase (HRP)-conjugated goat anti-rabbit IgG (1:5000, ZSGB-BIO, Beijing, China) and goat anti-mouse IgG (1:5000, ZSGB-BIO). The membranes were washed four times with PBST (10 min/wash). Immunolabeling was visualized by ECL solution (Pierce, IL, USA) and photographed by a gel image analysis system (Alpha Innotech, USA). Intensities were measured by FluorChem II software and relative intensities of target bands were analyzed and normalized to β-tubulin intensity.

### Planar microelectrode array recording

Rats were deeply anesthetized by intraperitoneal injection of pentobarbital sodium (40 mg/kg, Sigma) 2 h after BV administration and sacrificed by decapitation. The whole brain was carefully removed using Rongeurs and a spatula, and submerged in ice-cold artificial cerebrospinal fluid (aCSF containing in g/l: 6.838 NaCl, 0.268 KCl, 2.1 NaHCO_3_, 0.187 NaH_2_PO_4_ · 2H_2_O, 0.278 CaCl_2_, 0.244 MgCl_2_ · 6H_2_O, 1.98 glucose; pH 7.4, aerated with 95% O_2_ and 5% CO_2_ for 20 min before use). The cerebellum and brain stem were removed and the hemispheres separated. Two parallel cuts, rostral and ventral, were made at a ≈ 60° angle from the midline. A blocked hemisphere was mounted using super glue and sectioned transversely at 350 - 400 μm using a tissue slicer. The hippocampal-EC region was separated gently from slices and transferred to an incubation chamber containing carbogenated ACSF for 2 h before transfer to a recording chamber. The slice was secured with a slice anchor and perfused with carbogenated ACSF at 2 ml/min for recording.

Before electrophysiological recording using a MED-64 planar microelectrode array, [46], slices were photographed with a CCD camera (DP-70, OLYMPUS, Japan). The MED-P530A probe was connected to a 64-channel amplifier. Each probe corresponds to an amplifier channel and can deliver stimuli as well as record electrophysiological signals. One of the electrodes over the perforant pathway (PP) was used for stimulation. The electrode near the hippocampal sulcus producing the largest field excitatory postsynaptic potentials (fEPSPs) was chosen as the stimulating site for LTP experiments. A biphasic square-wave pulse (0.1 Hz, 0.2 ms, 10 - 199 μA) was delivered to evoke fEPSPs over the entire region underneath the array. Two input/output curves (fEPSP amplitude against stimulus intensity and fEPSP slope against stimulus intensity) were constructed from the means of five stimulus pulses at seven intensities (30, 60, 90, 120, 150, 180, and 199 μA). For induction of LTP, the stimulation intensity was set to elicit 50% of the maximum fEPSP according to the input/output curves. Following 30 min of stable baseline recording, LTP was induced by 10 trains, each comprised of 4 pulses at 100 Hz, with 200-ms inter-train intervals (θ burst stimulation, TBS). Post-TBS fEPSPs were recorded every 10 min for another 2 h. Sampling rate was 20 kHz and the signals were low-pass filtered at 0.1 Hz. All the stimulating protocols were generated by software.

### Open field test

To assess spontaneous locomotor activity, rats were tested in an open field (JLBehv-LAG-4, Shanghai Jiliang Software Technology) [47]. Each rat was gently placed in the center of the open field and allowed to explore freely. The spontaneous motor activity of 4 rats was simultaneously monitored using an automated camera-based computer tracking system. At the end of the test, the open field was cleaned with 75% ethyl alcohol. The open field was located in a room (22 - 26°C) isolated from sound and unintentional interruptions and lit by consistent background lighting. Each trial was conducted between 8:00 - 11:00 am and recorded for later analysis.

### Statistical analysis

Data are presented as mean ± S.E.M. Group means were compared using Mann-Whitney U-tests (for non-parametric data sets), paired-sample *t*-tests (for normally distributed data sets), and one-way analysis of variance (ANOVA) (for multi-group comparisons) with Fisher’s Protected Least Significant Difference (PLSD) test for post hoc pair-wise comparisons. All calculations were performed with SPSS software (SPSS Inc., Chicago, Il). A *P* < 0.05 was considered statistically significant.

## Abbreviations

TOR: Target of rapamycin; PIKK: Phosphoinositide 3-kinase related kinase; FKBP12: FK506-binding protein 12; 4EBP1: eIF4E binding protein-1; p70S6K: Ribosomal protein S6 kinase; eEF2: Eukaryotic elongation factor 2; LTP: Long-term potentiation; IASP: International Association for the Study of Pain; BV: Bee venom; RAPA: Rapamycin; ACSF: Artificial cerebrospinal fluid; DG: Dentate gyrus; PP: Perforant pathway; TBS: θ burst stimulation; HFS: High-frequency stimulation; fEPSP: Field excitatory postsynaptic potential; eIF4E: Eukaryotic initiation factor 4E.

## Competing interest

The authors declare that they have no completing interests.

## Authors’ contributions

DL, W-LY, NT, and Z-YZ collected most of the data reported here with contributions from FX and Y-JH; DL and NT conducted the Western blotting work; Z-YZ and FX conducted the open field test R-RW helped in MED64 system setup and slice preparation; JC and DL designed the experiments; H-YD and W-LY wrote the manuscript. All authors have read and approved the final version of the manuscript.
